# Invasive neurostimulation

**DOI:** 10.1186/1129-2377-16-S1-A29

**Published:** 2015-09-28

**Authors:** Marco Mercieri, Roberto Arcioni, Stefano Palmisani, Valentina Vano, Sara Tigano, Thomas Smith, Maria R Del Fiore, Barbara Silvestri, Adnan Al-Kaisy, Paolo Martelletti

**Affiliations:** Department of Medical-Surgical Sciences and Traslational Medicine, Sapienza University of Rome, Sant'Andrea Hospital, Rome, Italy; Pain Management & Neuromodulation Centre, Guy's & St Thomas’ NHS Foundation Trust, London, UK; Department of Clinical and Molecular Medicine, Sapienza University of Rome, Sant'Andrea Hospital, Rome, Italy

Chronic migraine afflicts 1-5% of the global population and poses a substantial burden on subjects’ quality of life and on health services utilization[1]. Although most patients benefit from abortive and preventive drugs, a subgroup of patients remains refractory to treatment. Refractory chronic migraine is one of the greatest challenges in headache medicine and, in these patients, invasive techniques should be considered.

In the past 20 years neuromodulatory approaches, already proved effective in other chronic pain syndromes, have been increasingly used for refractory primary headaches.

Neuromodulation, a reversible and adjustable manipulation of pain pathways is an evidence-based invasive treatment for chronic pain conditions and it may be applied to any neural structure: spinal cord, deep brain, and peripheral nerves.

Recently, three 12-week follow-up prospective, randomised trials have been conducted to validate occipital nerve stimulation in chronic migraine and intractable chronic migraine associated to occipital localization of pain. Considering the primary outcomes (50% reduction in pain intensity, 50% decrease of headache days) all the three trials have failed. In one of these studies[2], although the second follow-up at 52 weeks has shown important effects on pain severity, headache days, HIT-6 and MIDAS scores (60% of patients achieved 30% reduction in headache days and/or pain, 50% achieved 50% reduction in headache days and/or pain, 70% reported excellent or good headache relief and improved QoL, 70% would undergo the procedure again), it has also shown high incidence of adverse events related to the procedure (70% of patients experienced at least one AE, 41% of AEs required supplemental surgery, 8.6% of AEs required hospitalization).

A more recent prospective, open-label, exploratory study[3] assessing the long-term (6-months) safety, tolerability and efficacy of cervical high frequency (10 kHz), paresthesia-free, spinal cord stimulation in a cohort of 14 refractory chronic migraine patients (refractory also to Onabotulinumtoxin-A) has shown good results on reduction of headache days, medication intake, HIT-6 and MIDAS scores. The patients were carefully selected, for refractory chronic migraine, not considering topographic criteria for localization of pain, and were assessed by two different psychologists before eligibility. A significant reduction in headache days was observed at 24 weeks (average 7.0 days). Seven (50%) subjects recorded a >30% decrease in headache days, while 5 (36%) subjects reported a reduction in headache days greater than 50%. Eight subjects (57%) reverted to an episodic pattern of headache (<15 days a month). Medication intake reduced significantly, and four subjects discontinued triptans. Few adverse events have been reported.

HF10-SCS deserves further clinical investigations to evaluate its possible role in the management of rCM.
